# Supplementary material for: Different spectral sensitivities of ON- and OFF-motion pathways enhance the detection of approaching color objects in Drosophila
Source: Nat Commun. 2023 Nov 24;14:7693. doi: 10.1038/s41467-023-43566-8 (PMC10673857; doi:10.1038/s41467-023-43566-8)
Supplement: Supplementary file 1 — Supplementary Information [file 41467_2023_43566_MOESM1_ESM.pdf]

Supplementary Information

Different spectral sensitivities of ON- and OFF-motion pathways enhance the detection of approaching color objects in *Drosophila*

Kit D. Longden[1, 2], Edward M. Rogers[1], Aljoscha Nern[1], Heather Dionne[1], Michael B. Reiser[1,3].

[1]HHMI Janelia Research Campus, 19700 Helix Drive, Ashburn, VA 20176

[2]longdenk@janelia.hhmi.org; [3]reiserm@janelia.hhmi.org

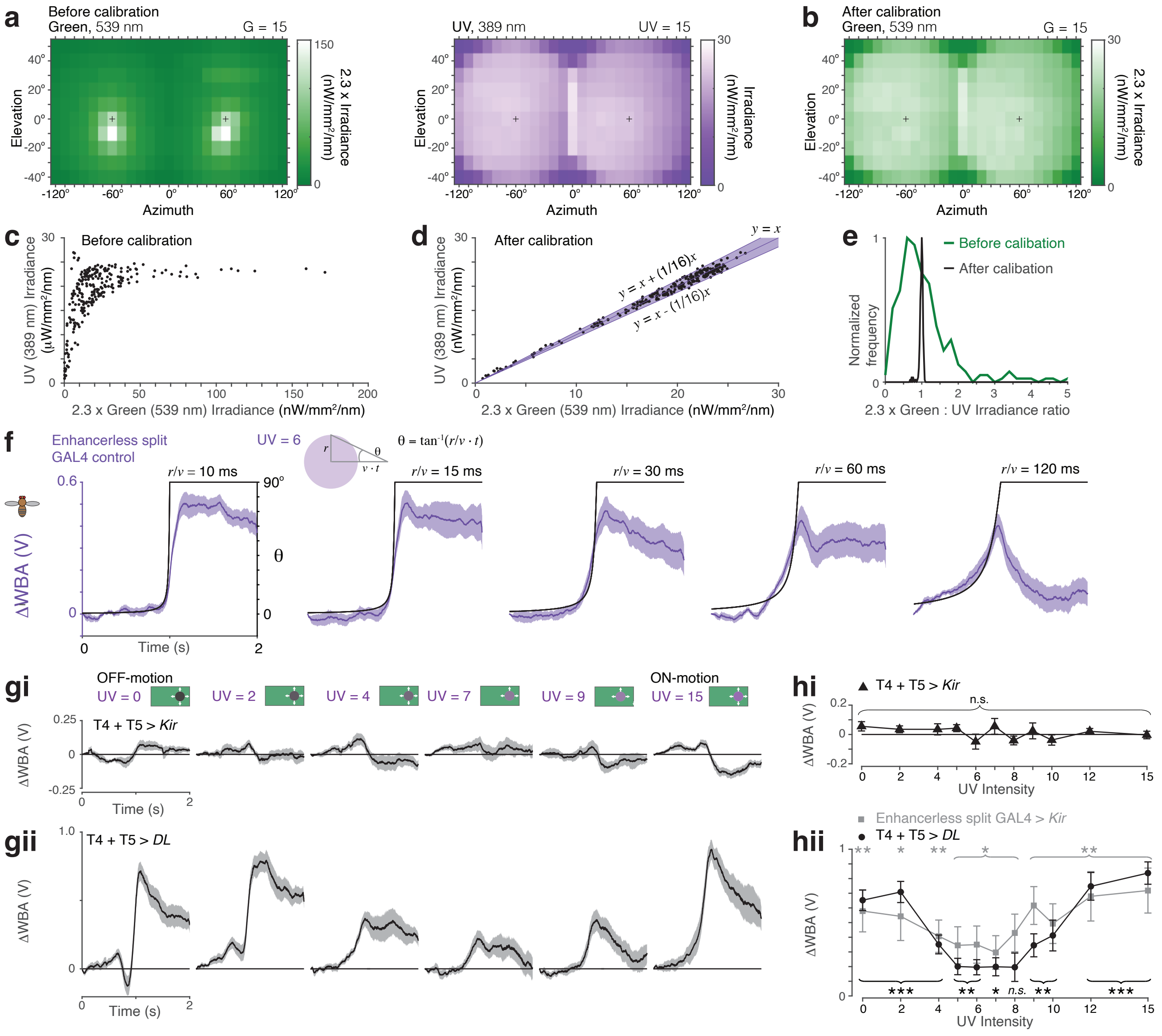

Supplementary Figure 1. Calibration of display system, speed tuning of the contribution of color circuitry to motion vision, and requirement of T4 and T5 cells. a. Spatial distributions across display screens of the irradiance of peak green and UV wavelengths prior to calibration (see Methods). The green irradiance was calibrated to a scaled factor of 2.3, relative to UV, as this resulted in behavior with isoluminance for *norpA*[36] Rh1-rescue flies in the mid-range of UV intensities. Black crosses at azimuth ±60° elevation 0° indicate foci of expanding discs. **b**. Spatial distributions of scaled irradiance of peak green wavelength after calibration. **c, d.** Distribution of all scaled green irradiance measurements plotted against UV irradiance, pre-calibration in (c), and post-calibration in (d). The irradiances were matched to the precision of the color bit depth, 1/16 (purple band). **e**. Histograms of ratio of green irradiance scaled by 2.3 and UV irradiance, for every location measured before and after calibration, corresponding to points plotted in panels (c) and (d). **f**. Responses of ES > *DL* control flies to approaching UV discs of different speeds, defined by r/v, with UV = 6. Black lines indicate the angle, θ, subtended by the radius of the disc. Mean ±SEM responses shown, N = 12 flies. **g**. Responses of flies with both T4 and T5 silenced by expression of $Kir_{2.1}$ (top, **gi**) compared to genetic control flies (bottom, **gii**). Mean ±SEM responses shown, N = 10 flies. Turning responses were abolished by the expression of $Kir_{2.1}$ in T4 and T5 for all intensities of UV, quantified in (h). **h**. Turning responses of flies

in (g), along with an additional control genotype, ES > *Kir*, measured as the response in the first 100 ms after the disc has fully expanded. Mean ±SEM responses shown, N = 10 flies. Two-sided student's t-test was used to identify responses significantly different from zero, with FDR correction for 11 comparisons. **hi**. Responses were not significantly different from zero with the expression of *Kir*$_{2.1}$ in T4 and T5 (p-values, left-to-right: 0.5, 0.5, 0.5, 0.5, 0.5, 0.5, 0.5, 0.7, 0.5, 0.5, 0.9). **hii**. Responses of controls were significantly different from zero (p-values, ES > *Kir*, left-to-right: 8E-3, 0.013, 9.8E-3, 0.026, 0.026, 0.029, 0.013, 6E-3, 9.8E-3, 8E-3, 6E-3; T4 + T5 > *DL*, left-to-right: 2E-5, 2E-5, 9E-4, 7E-3, 6E-3, 0.011, 0.07, 3E-3, 6E-3, 7E-5, 2E-5). Genotypes of all flies used in behavioral experiments are listed in Table 1. For all panels, asterisks indicate significance level: * p < 0.05, ** p < 0.01, *** p < 0.001, n.s. not significant. Source data are provided as a Source Data file.

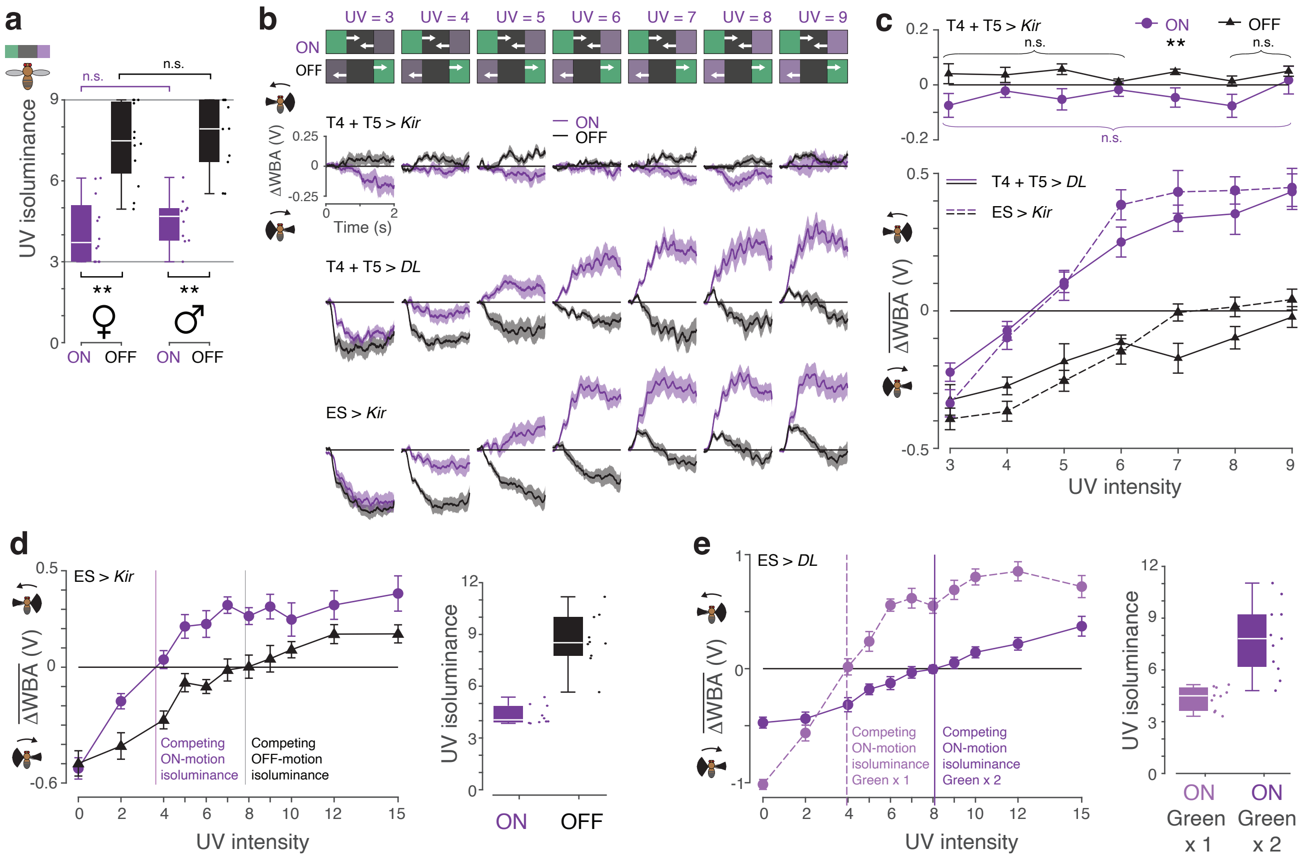

Supplementary Figure 2. Male and female behavioral responses to competing ON- and OFF-motion, the requirement of T4 and T5 cells, and effects of green intensity. a. Competing ON- and OFF-motion isoluminance levels of female (left) and male flies (right) measured in the replica setup. Neither competing ON- nor OFF-motion isoluminance levels were significantly different between the sexes (two-sided Wilcoxon signed rank test, N = 10 flies, ON p = 0.7, OFF p = 0.8), but were within each sex (two-sided Wilcoxon signed rank test, N = 10 flies, male p = 2E-3; female p = 3E-3). **b.** Direction-selective T4 and T5 neurons are required for responses to competing ON- (purple) and OFF-motion (black). Responses of flies with T4 and T5 silenced by expression of $Kir_{2.1}$ (top) and genetic control flies (middle, bottom) measured in the replica setup. To measure responses in the same flies, UV intensities were restricted to the range UV = 3–9. Mean ±SEM shown, N = 10 flies. **c**. Responses to competing ON- and OFF-motion of all flies with T4 and T5 silenced by expression of $Kir_{2.1}$ (top) and genetic control flies (bottom). Mean ±SEM shown, $N_{T4T5>Kir}$ =10, $N_{T4T5>DL}$ =10, $N_{ES>Kir}$ =13 flies. Turning responses were abolished by the expression of $Kir_{2.1}$ in T4 and T5 (two-sided student's t-test to identify responses significantly different from zero, with FDR correction for 7 comparisons; p-values for ON, left-to-right: 0.4, 0.5, 0.4, 0.6, 0.4, 0.4, 0.7; OFF, left-to-right: 0.3, 0.3, 0.06, 0.3, 6E-3, 0.5, 0.06); the exception was for UV = 7 for competing OFF-motion, where the response magnitude was small at 0.048 but significant (p = 6E-3). **d**. Left: Responses to competing ON- and OFF-motion of ES > *Kir* control flies over the full range 0 ≤ UV ≤ 15. Mean ±SEM shown, N = 10 flies. Right: isoluminance levels. **e**. To test the effect of the green luminance on the ON-motion isoluminance level, we doubled the green intensities used. Left: Responses to competing ON-motion of ES > *DL* control flies, with green intensity doubled (purple). Mean ±SEM shown, N = 11 flies. To aid comparisons, data from Fig. 2e is replotted (pale purple). Right: isoluminance levels. Genotypes of all flies used behavioral experiments are listed in Table 1. For all panels, boxplot conventions are as in Fig. 2d, and asterisks indicate significance level: * p < 0.05, ** p < 0.01, *** p < 0.001, n.s. not significant. Source data are provided as a Source Data file.

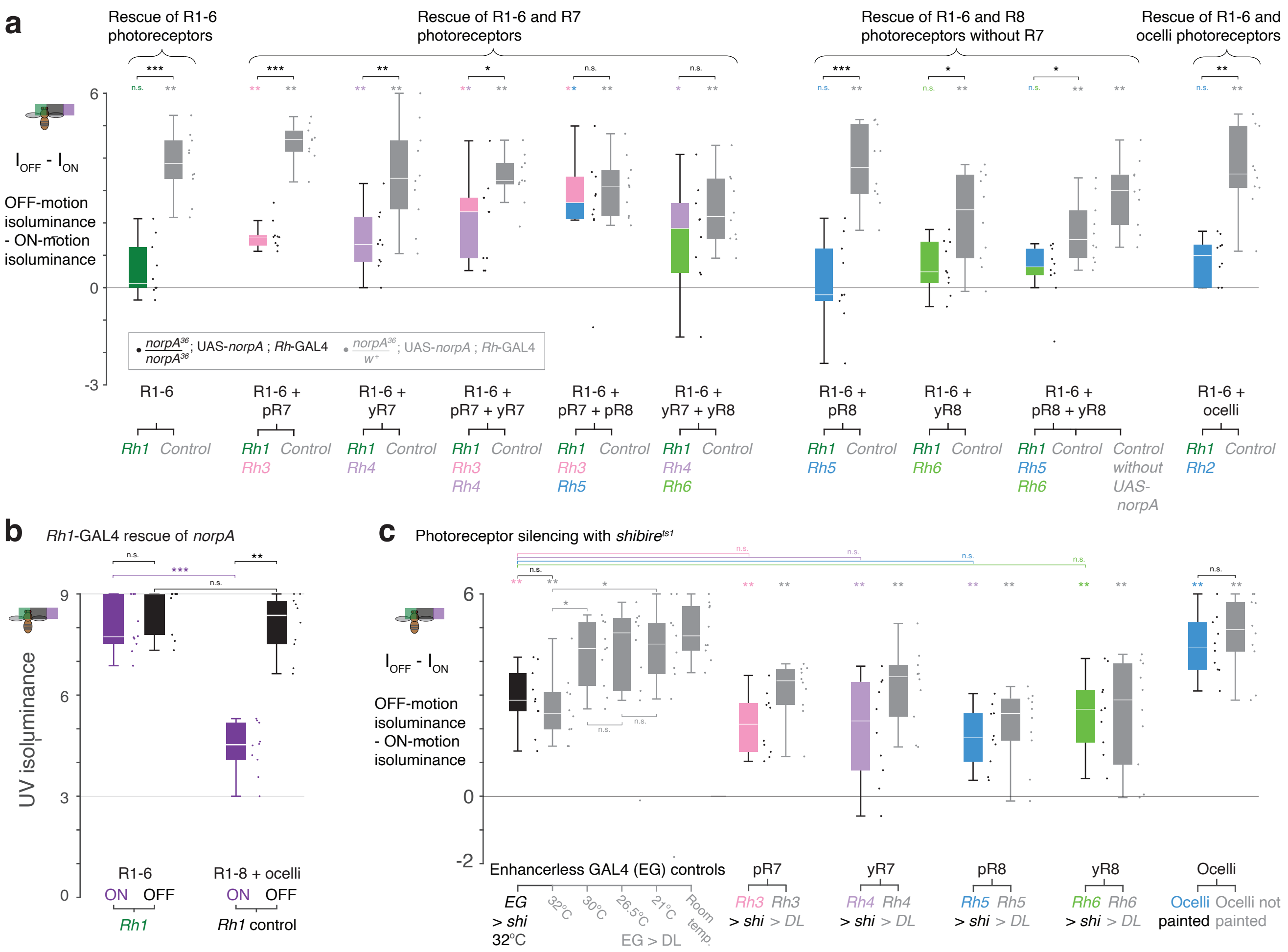

Supplementary Figure 3. Isoluminance levels for ON- and OFF-motion for genetic rescue and silencing experiments. a. Isoluminance levels for competing ON (I_{ON}, purple) and OFF-motion (I_{OFF}, black) of homozygous *norpA*^{36} flies with the function of different combinations of photoreceptors rescued using rhodopsin-GAL4 driven expression of UAS-*norpA*, along with I_{ON} (light purple) and I_{OFF} (gray) of genetic controls. The UV intensity was restricted to the range 3-9 to enable I_{ON} and I_{OFF} to be measured in the same flies. We used two-sided two-sample Wilcoxon rank sum tests to compare I_{ON} between rescue and control genotypes, with FDR correction for 9 comparisons, N = 10 flies (adjusted p-values: Rh1 Rh3, 5E-4 ; Rh1 Rh4, 1E-3; Rh1 Rh3 Rh4, 0.05; Rh1 R3 Rh5, 0.03; Rh1 Rh4 Rh6, 0.08; Rh1 Rh5, 5E-4; Rh1 Rh6 6E-4; Rh1 Rh5 Rh6, 3E-3; Rh1 Rh2, 5E-4), and to compare I_{OFF} between rescue and control genotypes, with FDR correction for 9 comparisons, N = 10 flies (adjusted p-values: Rh1 Rh3, 7E-3 ; Rh1 Rh4, 0.09; Rh1 Rh3 Rh4, 0.09; Rh1 R3 Rh5, 0.09; Rh1 Rh4 Rh6, 0.7; Rh1 Rh5, 0.3; Rh1 Rh6 0.5; Rh1 Rh5 Rh6, 0.4; Rh1 Rh2, 0.4). **b**. We silenced R1-6 using *Rh1*-GAL4 expression of UAS-*shibire*^{ts1} and heating the flies to 32°C. Plots show mean ±SEM turning responses for R1-6 silenced flies (*Rh1* > *shi* T = 32°C) and controls (*Rh1* > *shi* 21°C; *Rh1* > *DL* T = 21°C, 32°C). To compare responses between temperature conditions, we used two-sided two-sample Wilcoxon rank sum tests, with FDR correction for 7 comparisons, N = 10 flies (adjusted p-values, left-to-right: Rh1 > *shi* 32°C vs 21°C, ON 1E-3, 2E-3, 0.01, 0.1, 0.9, 0.01, 0.01; OFF 1E-3, 1E-3, 1E-3, 2E-3, 0.02, 0.5, 0.5; Rh1 > *DL* 32°C vs 21°C, ON 0.2, 0.044, 5E-3, 5E-3, 2E-3, 5E-3, 0.01; OFF 0.7, 0.7, 0.2, 0.7, 0.4, 0.9, 0.7). For R1-6 silenced flies (*Rh1* > *shi* T = 32°C), there was no significant difference

in the responses from the mean for competing ON-motion (left panel, two-sided student's t-test, FDR correction for 7 comparisons, N = 10 flies, adjusted p-values left-to-right: 0.7, 0.9, 0.7, 0.7, 0.7, 0.99, 0.98) or for OFF-motion (middle left panel, two-sided student's t-test, FDR correction for 7 comparisons, N = 10 flies, adjusted p-values left-to-right: 0.9, 0.9, 0.9, 0.9, 0.9, 0.9, 0.9). **c**. I_{ON} and I_{OFF} of flies with different classes of photoreceptors silenced by expressing UAS-*shibire*^{ts1} with rhodopsin GAL4 driver lines, and in EG control flies, along with genetic controls (based on same data as in Fig. 3c); for the ocelli, we used paint to block phototransduction. We used two-sided two-sample Wilcoxon rank sum tests to compare I_{ON} between silenced and control genotypes, with FDR correction for 7 comparisons, N = 10 flies (adjusted p-values: EG > *shi* T = 32°C and T = 21°C, 0.3; EG > *shi* and EG > *DL*, 0.3; *Rh3* > *shi* and *Rh3* > *DL*, 0.01; *Rh4* > *shi* and *Rh4* > *DL*, 0.02; *Rh5* > *shi* and *Rh5* > *DL*, 0.3; *Rh6* > *shi* and *Rh6* > *DL*, 0.3; Ocelli painted and unpainted, 0.3), and to compare I_{OFF} between silenced and control genotypes, with FDR correction for 7 comparisons, N = 10 flies (adjusted p-values: EG > *shi* T = 32°C and T = 21°C, 0.4; EG > *shi* and EG > *DL*, 0.2; *Rh3* > *shi* and *Rh3* > *DL*, 0.5; *Rh4* > *shi* and *Rh4* > *DL*, 0.7; *Rh5* > *shi* and *Rh5* > *DL*, 0.4; *Rh6* > *shi* and *Rh6* > *DL*, 0.3; Ocelli painted and unpainted, 0.8). Genotypes for all flies used in behavioral experiments are in Table 1. For all panels, boxplot conventions are as in Fig. 2d, and asterisks indicate significance level: * $p < 0.05$, ** $p < 0.01$, *** $p < 0.001$, n.s. not significant. Source data are provided as a Source Data file.

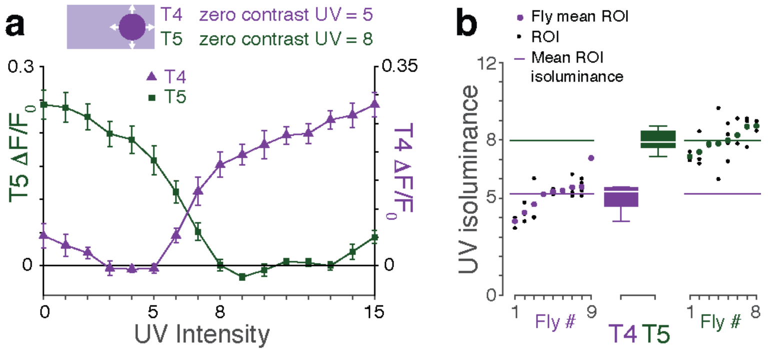

Supplementary Figure 4. Validation of isoluminance determination for T4 and T5 using contrast tuning. a. Calcium activity responses of T4 and T5 ROIs to UV discs expanding out of a UV background. For the T4 recordings, the background UV intensity was UV = 5, and so when the discs shared this intensity, they were unambiguously isoluminant. For the T5 recordings, the background UV intensity was UV = 8, and so when the discs shared this intensity, they were unambiguously isoluminant. We chose these background intensities to test our methods at comparable illuminance levels to the values determined from the experiments with UV discs expanding out of a green background (Fig. 4f). Mean ±SEM shown, $N_{\mathrm{T4,\,flies}} = 9$, $N_{\mathrm{T5,\,flies}} = 8$, different flies to those in Fig. 4. **b**. Isoluminance levels of ROIs (black dots) and flies (colored circles) for T4 (purple) and T5 (green) cells. Colored horizontal lines indicate the mean ROI isoluminances: 5.2 ±0.3 for T4 (mean ±SEM, $N_{\mathrm{T4,\,flies}} = 9$), and 7.7 ±0.3 for T5 (mean ±SEM, $N_{\mathrm{T5,\,flies}} = 8$). Genotypes for all flies used in imaging experiments are in Table 2. Boxplot conventions are as in Fig. 2d. Source data are provided as a Source Data file.

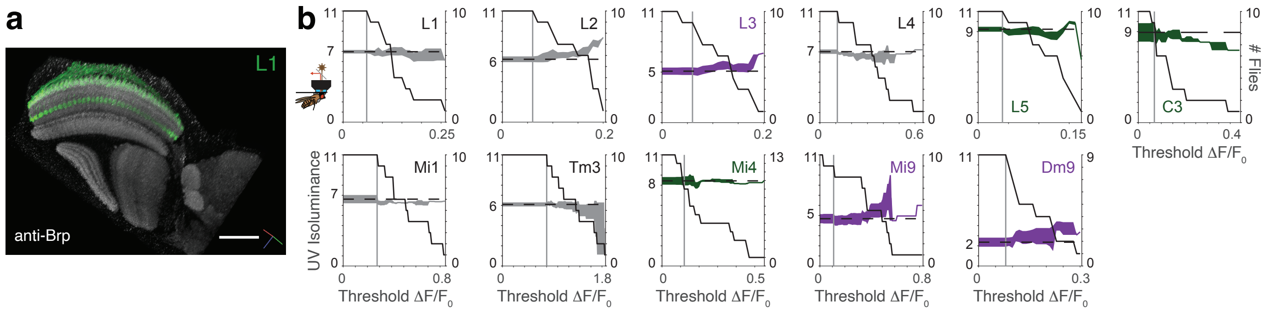

Supplementary Figure 5. Imaging of T4 input cells, lamina cells and Dm9. **a**. Horizontal section through the optic lobe visualizing the expression pattern (green) for the L1 split GAL4 line showing distinguishing expression in medulla layers 1 and 5 (see Fig. 5a). Gray is nc82 antibody staining for Bruchpilot to indicate neuropils. Scale bar: 50µm. **b**. Mean ROI isoluminance levels of all cell types when the threshold for excluding non-visual ROIs is varied, mean ±SEM across flies indicated by color line and shading, and black lines indicate the number of flies. Vertical gray lines indicate the thresholds used to exclude non-responding ROIs, and horizontal dashed lines indicate the corresponding mean ROI isoluminance levels calculated. Genotypes for all flies used in imaging experiments are in Table 2. Source data are provided as a Source Data file.

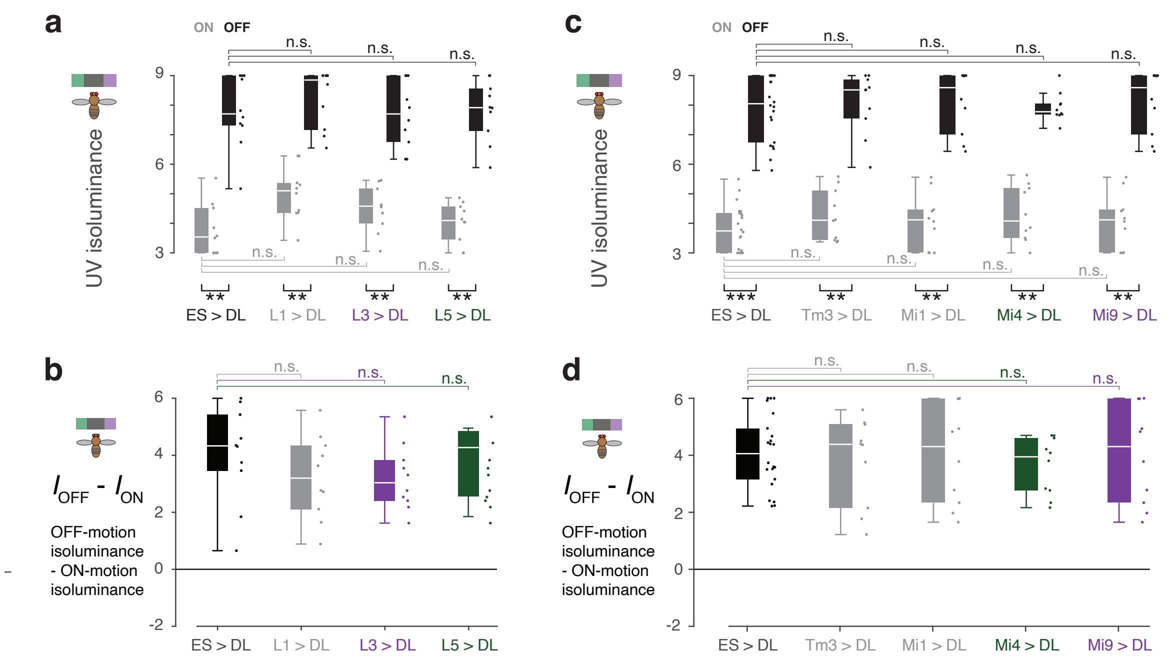

Supplementary Figure 6. UV-sensitivity of behavioral responses to ON- and OFF-motion in wild type controls. **a**. I_{ON} (gray) and I_{OFF} (black) for flies with split GAL4 driver lines selective for LMC cell types L1, L3 and L5 and control (ES > *DL*) flies, using the compact protocol where I_{ON} and I_{OFF} were measured in the same flies. As in Fig. 6, we used one-sided Wilcoxon rank sum test to compare whether I_{ON} were greater than controls, or I_{OFF} were less than controls, with FDR correction for 3 comparisons, $N_{L1 > DL, \text{ flies}} = N_{L3 > DL, \text{ flies}} = N_{L5 > DL, \text{ flies}} = 10$, $N_{ES > DL, \text{ flies}} = 12$ (adjusted p-values for I_{ON}: L1 > *DL* 0.2, L3 > *DL* 0.3, L5 > *DL* 0.5; for I_{OFF}: L1 > *DL* 0.9, L3 > *DL* 0.9, L5 > *DL* 0.9). We used two-sided paired Wilcoxon signed rank test to compare isoluminance levels within genotypes (p-values: ES > *DL* 3E-3; L1 > *DL* 2E-3; L3 > *DL* 2E-3; L5 > *DL* 2E-3). **b**. Pairwise differences between the I_{ON} and I_{OFF} of flies shown in panel (a). We used one-sided Wilcoxon rank sum test to compare whether ON responses were greater than controls, or OFF responses were less than controls, with FDR correction for three comparisons (adjusted p-values: L1 > *DL* 0.6, L3 > *DL* 0.6, L5 > *DL* 0.7). **c**. I_{ON} and I_{OFF} for flies with split GAL4 driver lines selective for T4 input cell types Mi1, Tm3, Mi4 and Mi9 and genetic controls (ES > *DL*). Statistical tests as in panel (a), with FDR correction for 4 comparisons, $N_{Mi1 > DL, \text{ flies}} = N_{Tm3 > DL, \text{ flies}} = N_{Mi4 > DL, \text{ flies}} = N_{Mi9 > DL, \text{ flies}} = 10$, $N_{ES > DL, \text{ flies}} = 22$ (adjusted p-values for I_{ON}: Tm3 > *DL* 0.2, Mi1 > *DL* 0.2, Mi4 > *DL* 0.2, Mi9 > *DL* 0.2; for I_{OFF}: Tm3 > *DL* 0.7, Mi1 > *DL* 0.7, Mi4 > *DL* 0.7, Mi9 > *DL* 0.7; within genotype comparison p-values: ES > *DL* 4E-5; Tm3 > *DL* 2E-3; Mi1 > *DL* 2E-3; Mi4 > *DL* 2E-3; Mi9 > *DL* 2E-3). **d**. Pairwise differences between I_{OFF} and I_{ON} of flies shown in panel (c). Statistical tests as in panel (b), with FDR correction for 4 comparisons (adjusted p-values: Tm3 > *Kir* 0.5, Mi1 > *Kir* 0.5, Mi4 > *Kir* 0.5, Mi9 > *Kir* 0.5). Genotypes for all flies used in behavioral experiments are in Table 1. For all panels, asterisks indicate significance level: * p < 0.05, ** p < 0.01, *** p < 0.01, n.s. not significant, and boxplot conventions are as in Fig. 2d. Source data are provided as a Source Data file.
