# Supplementary material for: Different spectral sensitivities of ON- and OFF-motion pathways enhance the detection of approaching color objects in Drosophila
Source: Nat Commun. 2023 Nov 24;14:7693. doi: 10.1038/s41467-023-43566-8 (PMC10673857; doi:10.1038/s41467-023-43566-8)
Supplement: Supplementary file 3 — Reporting Summary [file 41467_2023_43566_MOESM3_ESM.pdf]

Corresponding author(s): Michael Reiser

Last updated by author(s): Oct 27, 2023

Reporting Summary

Nature Portfolio wishes to improve the reproducibility of the work that we publish. This form provides structure for consistency and transparency in reporting. For further information on Nature Portfolio policies, see our Editorial Policies and the Editorial Policy Checklist.

Statistics

For all statistical analyses, confirm that the following items are present in the figure legend, table legend, main text, or Methods section.

n/a	Confirmed	
☐	☒	The exact sample size (n) for each experimental group/condition, given as a discrete number and unit of measurement
☐	☒	A statement on whether measurements were taken from distinct samples or whether the same sample was measured repeatedly
☐	☒	The statistical test(s) used AND whether they are one- or two-sided<br>*Only common tests should be described solely by name; describe more complex techniques in the Methods section.*
☐	☒	A description of all covariates tested
☐	☒	A description of any assumptions or corrections, such as tests of normality and adjustment for multiple comparisons
☐	☒	A full description of the statistical parameters including central tendency (e.g. means) or other basic estimates (e.g. regression coefficient) AND variation (e.g. standard deviation) or associated estimates of uncertainty (e.g. confidence intervals)
☐	☒	For null hypothesis testing, the test statistic (e.g. F, t, r) with confidence intervals, effect sizes, degrees of freedom and P value noted<br>*Give P values as exact values whenever suitable.*
☐	☒	For Bayesian analysis, information on the choice of priors and Markov chain Monte Carlo settings
☐	☒	For hierarchical and complex designs, identification of the appropriate level for tests and full reporting of outcomes
☐	☒	Estimates of effect sizes (e.g. Cohen's d, Pearson's r), indicating how they were calculated

Our web collection on statistics for biologists contains articles on many of the points above.

Software and code

Policy information about availability of computer code

Data collection	MATLAB data acquisition toolbox (v3.14) used for behavioral data acquisition.<br>Psychophysics toolbox (v3.0.15; Brainard, D. H. 1997. Spat. Vis. 10, 433–436) for displaying visual patterns.<br>Prairie View (v5.4) for calcium imaging data acquisition
Data analysis	MATLAB 2018b for data anlaysis and plotting (custom code to be shared at https://github.com/reiserlab/<br>V3D (Peng et al. 2010. VNat. Biotechnol. 28, 348–353) for generating resampled views for immunohistochemistry.

For manuscripts utilizing custom algorithms or software that are central to the research but not yet described in published literature, software must be made available to editors and reviewers. We strongly encourage code deposition in a community repository (e.g. GitHub). See the Nature Portfolio guidelines for submitting code & software for further information.

Data

Policy information about availability of data

All manuscripts must include a data availability statement. This statement should provide the following information, where applicable:

- Accession codes, unique identifiers, or web links for publicly available datasets
- A description of any restrictions on data availability
- For clinical datasets or third party data, please ensure that the statement adheres to our policy

The processed data generated used in this study have been deposited in the Zenodo database with DOI 10.5281/zenodo.10045303. Raw data are available on

request from the corresponding author. Source data are provided with this paper. MATLAB code for plotting the figures from source data has been deposited in the Zenodo database with DOI 10.5281/zenodo.10045303.

Human research participants

Policy information about studies involving human research participants and Sex and Gender in Research.

Reporting on sex and gender	N/A
Population characteristics	N/A
Recruitment	N/A
Ethics oversight	N/A

Note that full information on the approval of the study protocol must also be provided in the manuscript.

Field-specific reporting

Please select the one below that is the best fit for your research. If you are not sure, read the appropriate sections before making your selection.

☒ Life sciences ☐ Behavioural & social sciences ☐ Ecological, evolutionary & environmental sciences

For a reference copy of the document with all sections, see nature.com/documents/nr-reporting-summary-flat.pdf

Life sciences study design

All studies must disclose on these points even when the disclosure is negative.

Sample size	Sample sizes were chosen without calculations based on typical sample sizes used in prior work to allow for statistical analysis. For behavioral experiments, 10 flies were recorded with 5 trials each; the exception was the measurements of experiments with different speeds (Supplementary Fig. 1f) where there were 7 flies with 5 trials each. For imaging experiments, there were at least 9 flies per cell type, with between 2 and 6 ROIs per fly. These sample sizes are typical for similar studies.
Data exclusions	No data were excluded from analysis, except when flies stopped flying during behavioral tests, and when brain movement was large during imaging experiments. The procedures for classifying visually responsive and unresponsive ROIs are described in detail, and the consequences of thresholds are plotted in Fig. 4j and Supplementary Fig. 5b.
Replication	The UV/green projector setup was replicated, and results were replicated on this second setup. All attempts at replication were successful. Further details of replication in the Methods.
Randomization	Experimental groups were determined by the genotype of the flies, flies of different genotypes were not randomized but recorded during similar time frames using the same experimental protocol. Stimulus presentation trials were randomized.
Blinding	The investigators were not blinded to the fly genotypes. The same experimental procedures were applied to all groups being compared within the dataset.

Reporting for specific materials, systems and methods

We require information from authors about some types of materials, experimental systems and methods used in many studies. Here, indicate whether each material, system or method listed is relevant to your study. If you are not sure if a list item applies to your research, read the appropriate section before selecting a response.

Materials & experimental systems

n/a	Involved in the study	
☐	☒	Antibodies
☒	☐	Eukaryotic cell lines
☒	☐	Palaeontology and archaeology
☐	☒	Animals and other organisms
☒	☐	Clinical data
☒	☐	Dual use research of concern

Methods

n/a	Involved in the study	
☒	☐	ChIP-seq
☒	☐	Flow cytometry
☒	☐	MRI-based neuroimaging

Antibodies

Antibodies used	We use a widely used antibodies for GFP and Brp as a general neuropile label. Antibody dilutions were as follows: rabbit anti-GFP,1:1000; mouse anti-Brp (nc82), 1:30.
Validation	All antibodies used (and associated labeling protocols) have been shown to be effective for our purpose (Drosophila neuroanatomy) in dozens of prior studies.

Animals and other research organisms

Policy information about studies involving animals; ARRIVE guidelines recommended for reporting animal research, and Sex and Gender in Research

Laboratory animals	Drosophila melanogaster (DL wild type strain and many lab strains, detailed in Tables 1-2), Drosophila mauritiana, Drosophila sechellia, Drosophila santomea, Drosophila yakuba, and Drosophila teissieri (from strains maintained by the Stern Lab, at HHMI Janelia). Females 2-5 days old post-eclosion were used.
Wild animals	This study did not involve wild animals.
Reporting on sex	Most experiments, except where indicated, were carried out on female flies.
Field-collected samples	This study did not involve samples collected from the field.
Ethics oversight	No ethical approval was required for experiments with Drosophila flies.

Note that full information on the approval of the study protocol must also be provided in the manuscript.
